# Continuous subcutaneous insulin infusion alters microRNA expression and glycaemic variability in children with type 1 diabetes

**DOI:** 10.1038/s41598-021-95824-8

**Published:** 2021-08-17

**Authors:** Emma S. Scott, Andrzej S. Januszewski, Luke M. Carroll, Gregory R. Fulcher, Mugdha V. Joglekar, Anandwardhan A. Hardikar, Timothy W. Jones, Elizabeth A. Davis, Alicia J. Jenkins

**Affiliations:** 1 NHMRC Clinical Trials Centre, Faculty of Medicine and Health, University of Sydney, Sydney, Australia; 2 Department of Endocrinology and Diabetes, Royal North Shore Hospital, Sydney, Australia; 3 Department of Medicine, St Vincent’s Hospital, University of Melbourne, Melbourne, Australia; 4 Northern Clinical School, University of Sydney, Sydney, Australia; 5 University of Western Australia, Perth, Australia; 6 Diabetes and Endocrinology Services, Perth Children’s Hospital, Perth, Australia; 7 Telethon Kids Institute, Perth, Australia; 8 NHMRC Clinical Trials Centre, The University of Sydney, Locked bag, 77, Camperdown, NSW 1450 Australia

**Keywords:** Diabetes, Type 1 diabetes

## Abstract

To determine whether continuous subcutaneous insulin infusion (CSII) vs. multiple daily injections (MDI) therapy from near-diagnosis of type 1 diabetes is associated with reduced glycaemic variability (GV) and altered microRNA (miRNAs) expression. Adolescents (74% male) within 3-months of diabetes diagnosis (n = 27) were randomized to CSII (n = 12) or MDI. HbA1c, 1-5-Anhydroglucitol (1,5-AG), high sensitivity C-peptide and a custom TaqMan qPCR panel of 52 miRNAs were measured at baseline and follow-up (median (LQ-UQ); 535 (519–563) days). There were no significant differences between groups in baseline or follow-up HbA1c or C-peptide, nor baseline miRNAs. Mean ± SD 1,5-AG improved with CSII vs. MDI (3.1 ± 4.1 vs. − 2.2 ± − 7.0 mg/ml respectively, *P* = 0.029). On follow-up 11 miRNAs associated with diabetes vascular complications had altered expression in CSII-users. Early CSII vs. MDI use is associated with lower GV and less adverse vascular-related miRNAs. Relationships with future complications are of interest.

## Introduction

In Type 1 diabetes continuous subcutaneous insulin infusion (CSII) use is associated with reduced chronic complications compared to multiple daily insulin injections (MDI)^1^. Mechanisms may relate to reduced insulin dose, glycaemic variability (GV), inflammation and oxidative stress. Circulating microRNAs (miRNAs) released in response to cellular damage may be biomarkers of diabetes and its complications^2^. The impact of early CSII therapy on miRNA expression in type 1 diabetes has not been investigated. We assess whether CSII compared to MDI therapy within 3-months of type 1 diabetes diagnosis is associated with differences in glycaemia, GV, C-peptide, and miRNA expression.

## Methods

Adolescents (n = 27) at an Australian tertiary referral paediatric diabetes clinic were randomised via block randomization to CSII or MDI therapy within 3-months after diagnosis of type 1 diabetes. Baseline blood samples were taken (median (LQ-UQ)) 1 (1–21) days post-diagnosis, prior to randomisation to CSII (n = 12) or MDI (n = 15) treatment and at 1–3 subsequent time-points, at which HbA1c, and C-peptide (high sensitivity TOSOH AIA-600 and Roche Modular assays) were measured and sera stored for 1,5-anhydroglucitol (1,5-AG), GlycoMark (Winston-Salem, NC)^3^ and miRNAs. Mean differences, standard deviation (SD) and coefficient of variation (CV) of HbA1c and 1,5-AG were compared between treatment arms. No continuous glucose monitoring (CGM) was performed. We previously conducted a plasma miRNA discovery analysis in 30 adults with type 1 diabetes (15 with and 15 without microvascular complications) and 15 matched normoglycaemic individuals. The discovery analysis has been presented at the American Diabetes Association Scientific Meeting^4^. The 52 significantly different miRNAs identified during the discovery screen were measured herein (custom TaqMan miRNA qPCR OpenArray panels). Briefly, RNA was isolated using Trizol (Thermo Scientific) on a (QiaCube-HT robot, with RNeasy-HT kit (Qiagen) and ath-miR-172a (spike-in control). RNA concentration was then quantified (Eon spectrophotometer, Biotek, Winooski, VT). Reverse transcription (ath-miR-159a spike-in control), pre-amplification and OpenArray qPCR for microRNAs used a low-sample input workflow^5^. MiRNA expression was normalised to spike-in miRNA controls. Data were analysed using IBM SPSS Statistics (version 23) and Graph Pad Prism (v6.0), including: descriptive statistics, paired and independent T-tests, Mann–Whitney U, Chi-square and Spearman rank correlation tests, with significance at *p* < 0.05.

### Ethics approval

Ethics approval for the study was granted by the Department of Health WA Human Research Ethics Committee HREC 2016155EP, participants were consented to participation and publication of data. The datasets analysed during the current study are not publicly available due to lack of consent from study participants and lack of clearance from the appropriate human ethics committee. The research was done in accordance with the relevant guidelines and performed in accordance with the Declaration of Helsinki.

## Results

There were no significant differences in baseline characteristics between CSII and MDI groups: (mean (SD)) age 14.1 (1.3) years, male (n (%)) 20 (74), BMI (median (IQR)) 20.0 (18.9–21.6) kg/m^2^, HbA1c median (IQR) 12.4 (10.3–14.0)% (112 (89–130) mmol/mol) or 1-5-AG (median (IQR)) 3.1 (2.0–5.7) μg/ml). Both groups had similar C-peptide at baseline and study-end (follow-up median (LQ-UQ) (535 (519–63) days)). There were no group differences in HbA1c at baseline (above) or follow-up, nor change over follow-up (mean ± SD; − 4.6 ± 2.4% (− 50 ± 26 mmol/mol) CSII vs. − 3.5 ± 2.8% (− 38 ± 31 mmol/mol) MDII, *P* = 0.3). There were no differences in HbA1c CV or SD between groups. 1,5-AG increased (improved) with CSII (3.1 ± 4.1 mg/ml CSII vs. − 2.2 ± 7.0 mg/ml MDI, *P* = 0.029), which remained significant after adjustment for follow-up duration. There were no significant differences in baseline miRNA expression by treatment modality, however, 11 miRNAs had significantly altered expression (measured between the baseline and end of follow up) in CSII- vs. MDI-users (Table 1). Baseline, endpoint miRNA expression or change in miRNA expression did not correlate with changes in 1,5-AG levels.

**Table 1:** Fold difference in microRNA expression CSII compared to MDI therapy at study end-point.

microRNA	Cycle threshold (CT) (mean)			Fold difference expression^¶^	*P*-value
	CSII (end point—baseline)	MDI (end point—baseline)	CSII-MDI		
**Increased expression (both arms) compared to baseline**					
miR-106a	− 3.58	− 9.99	6.41^§^	85	**0.026**
miR-483-5p	− 6.92	− 10.93	4.01^§^	16	**0.048**
**Reduced expression (both arms) compared to baseline**					
miR-24	6.08	0.39	5.79^‡^	55	**0.0089**
miR-21	10.45	4.30	6.15^‡^	71	**0.036**
miR-328	9.75	2.53	7.22^‡^	149	**0.042**
**Differing expression (increased in one; reduced in other arm) compared to baseline**					
miR-99b	1.42	− 12.44	13.86^†^	14,885	**0.0048**
miR-320	0.75	− 11.67	12.42^†^	5474	**0.011**
miR-375	1.47	− 5.88	7.35^†^	163	**0.019**
miR-28	2.96	− 5.64	8.59^†^	386	**0.026**
miR-17	3.48	− 4.17	7.65^†^	201	**0.031**
miR-92a	2.20	− 9.67	11.87^†^	3746	**0.042**

Positive endpoint—baseline value indicates higher CT level (lower expression) at the end of follow-up. Negative value indicates lower CT level (higher expression) at the end of follow-up relative to baseline.

^†^Expression at endpoint relative to baseline reduced in CSII, and increased in MDI.

^‡^Expression at endpoint relative to baseline reduced in CSII, smaller reduction in MDI.

^§^Expression at endpoint relative to baseline increased in CSII, greater increase in MDI.

^¶^Calculated as 2^(CSII-MDI).

Significant *P*-values are highlighted in bold.

## Discussion

This study demonstrates that early CSII use in youth with type 1 diabetes is associated with a better 1,5-AG compared to MDI, implying reduced GV with CSII therapy. Complications-related miRNA expression over a median 1.5-years follow-up differed by insulin delivery modality.

CSII has been demonstrated to have a greater HbA1c benefit than MDI therapy in meta-analyses, including in adolescents^6^. Epidemiological studies report reduced complications and mortality with CSII vs. MDI use^1, 6^. CSII with continuous glucose monitoring is associated with even greater HbA1c benefits^7^. As yet there are no long-term data regarding glucose-sensor enabled CSII and chronic complications. This study did not demonstrate HbA1c benefit with CSII use, similar to a recent paediatric type 1 diabetes UK trial^8^. Lack of HbA1c differences may relate to the follow-up duration, or added learning associated with recent-onset diabetes and CSII therapy. We found no impact of treatment modality on C-peptide, in keeping with the Pediatric Onset Study, where initial C-peptide benefits of sensor-augmented pump therapy were not sustained at 2-years^9^.

We demonstrated CSII use to be associated with a more favorable increase in 1,5-AG levels than MDI. 1,5-AG reflects glycaemia over weeks, and correlates with markers of GV including glucose SD and mean amplitude of glycaemic excursion (MAGE)^3^. Higher GV is associated with increased risk of chronic complications^10^, thus relationships between early CSII therapy and subsequent chronic complications merits exploration.

Levels of baseline miRNAs related to microvascular complications in adults with type 1 diabetes did not differ by insulin delivery modality, but diverged during follow-up, with significant differences in the change in expression (between baseline and study end) in levels of 11 miRNAs (Table 1). As HbA1c and C-peptide changed similarly in both CSII and MDI users, miRNA differences more likely reflect true insulin delivery modality differences rather than HbA1c or residual C-peptide effects. This may relate to CSII associated lower GV, inflammation or oxidative stress.

In the EURODIAB Prospective Complications Study, miR-106a and miR-17 were higher in pooled sera of type 1 diabetes subjects with vs. without vascular complications^11^. At our study-end relative to baseline, miR-17 expression decreased with CSII use and increased in MDI users. miR-106a levels increased with both modalities, but to a significantly greater level with MDI. EURODIAB described downregulation of miR-483-5p, miR-92a, miR-320, and miR-24 with complications. We observed that miR-24 decreased with both treatments, more so with CSII; miR-92a and miR-320 decreased with CSII and increased with MDI. miR-483-5p increased with both modalities. In the DIabetic REtinopathy Candesartan Trials miR-320a was downregulated with nephropathy^2^. Several groups report upregulated miR-21 in individuals with both nephropathy and retinopathy^2^. Increased miR-21, is also associated with high GV in cultured cells^12^. miR-21 levels fell to a greater extent over follow-up in our CSII vs. MDI users. miR-28 expression reduced from baseline in CSII users and increased in the MDI group. miR-28 has been associated with reduced risk of microalbuminuria in the Pittsburgh Epidemiology of Diabetes Complications (EDC) study^2^. We observed reduced miR-99b relative to baseline in the CSII group, whereas expression increased compared to baseline with MDI. miR-99b was increased in urinary samples of individuals with albuminuria in the EDC study^13^. miR-328 expression reduced compared to baseline in both the CSII and MDI group, however the degree of reduction was less in MDI users. miR-328 has been associated with impaired wound healing in diabetes^14^. Relative to baseline, levels of miR-375, associated with beta-cell death^15^, fell in CSII users and increased in MDI users, but did not correlate with C-peptide changes.

Study strengths are the longitudinal randomised trial design, inclusion of 1,5AG and miRNAs assessed by sensitive TaqMan PCR chemistry, and lack of group differences in baseline parameters and follow-up HbA1c and C-peptide. Limitations include small sample size, lack of CGM, inflammation and oxidative stress data, and lack of long-term data regarding complications and need for further knowledge relating miRNAs, each of which impacts multiple genes, to clinical outcomes.

CSII therapy from near diagnosis of type 1 diabetes in youth is associated with lower GV than MDI therapy and an altered pattern of microRNA expression. The latter may be an early signal of better vascular health in CSII-treated youth. Replication and mechanistic studies and long-term follow-up are merited.
